# pyPept: a python library to generate atomistic 2D and 3D representations of peptides

**DOI:** 10.1186/s13321-023-00748-2

**Published:** 2023-09-12

**Authors:** Rodrigo Ochoa, J. B. Brown, Thomas Fox

**Affiliations:** Medicinal Chemistry, Boehringer Ingelheim Pharma GmbH & Co KG, 88397 Biberach/Riss, Germany

**Keywords:** Peptide, Python, Conformer, BILN, RDKit, Cheminformatics, Molecule depiction

## Abstract

We present pyPept, a set of executables and underlying python-language classes to easily create, manipulate, and analyze peptide molecules using the FASTA, HELM, or recently-developed BILN notations. The framework enables the analysis of both pure proteinogenic peptides as well as those with non-natural amino acids, including support to assemble a customizable monomer library, without requiring programming. From line notations, a peptide is transformed into a molecular graph for 2D depiction tasks, the calculation of physicochemical properties, and other systematic analyses or processing pipelines. The package includes a module to rapidly generate approximate peptide conformers by incorporating secondary structure restraints either given by the user or predicted via pyPept, and a wrapper tool is also provided to automate the generation and output of 2D and 3D representations of a peptide directly from the line notation. HELM and BILN notations that include circular, branched, or stapled peptides are fully supported, eliminating errors in structure creation that are prone during manual drawing and connecting. The framework and common workflows followed in pyPept are described together with illustrative examples. pyPept has been released at: https://github.com/Boehringer-Ingelheim/pyPept.

**Graphical Abstract:**

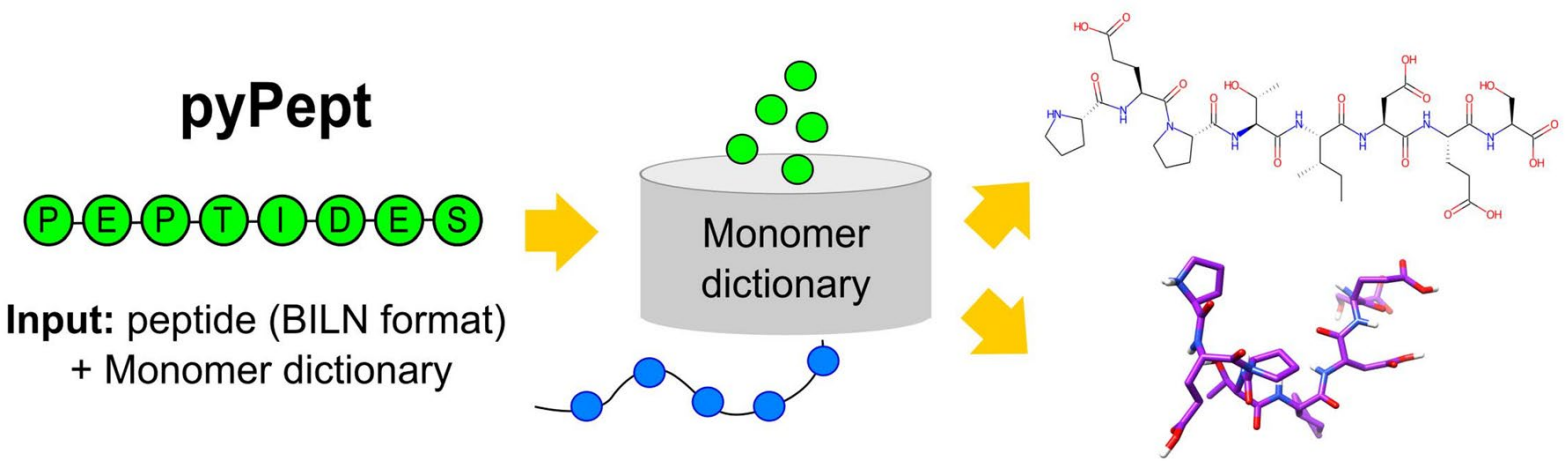

**Supplementary Information:**

The online version contains supplementary material available at 10.1186/s13321-023-00748-2.

## Introduction

Peptides as therapeutic or diagnostic agents are a modality with proven translational success in clinical applications; more than 80 drugs on the market are peptide molecules, with others actively in clinical trials [1]. In an accompanying fashion, suitable in silico tools to represent, process, and analyze peptides have been steadily published [2–5].

Among the available open source tools, many are restricted to natural amino acids, but some also support enhancement by a selected set of non-natural amino acids (NNAAs). This is the case for packages available in the Rosetta Commons project [6, 7], the PEPstrMOD webserver [8], and the SwissSideChain database [9]. Some tools, inspired by full proteins, can fail when dealing with more complex peptidic structures, including staples (e.g., a hydrocarbon chain attached to two amino acids in order to help maintain alpha-helical conformation), non-stapled cyclic peptides, or multi-chain branched peptides [10].

For the representation of peptides, the FASTA format is widely used and can be employed, e.g., to calculate peptide properties based on experimentally available physicochemical properties of natural monomers [11]. However, it is restricted to natural amino acids and simple peptides without extra bonds such as those involved in formation of cyclic peptides. For example, the natural hormone oxytocin can be represented in FASTA by its sequence CYIQNCPLG, but the ring formed by the disulfide bond between the first and sixth cysteine monomers is not accounted for.

For more complex macromolecular entities, line notations that go beyond the simple FASTA format have been developed. A prominent example is the Hierarchical Editing Language for Macromolecules (HELM) [12, 13]. It relies on a monomer library that defines the individual monomers and their possible connection points. Together with the information on how these monomers are connected, this allows an unambiguous representation of even very complex biomolecular entities in a string. Recently, we described an intuitive line notation termed BILN (Boehringer Ingelheim Line Notation) [14], where a simple but, critically, human-readable and robust format allows the representation and manipulation of complex multi-chain peptides including staples and cycles.

In principle, small molecule cheminformatics tools are also applicable to peptides [15]. However, they look at the peptide as a whole and neglect its construction principle that it is an ordered sequence of monomers, something that potentially could be used for more efficient computations. In addition, even when these tools offer monomer support, this is usually limited to a hard-coded list of amino acids. RDKit, a widely used open-source package for cheminformatics tasks [16], contains functions to process HELM inputs, but only for a set of 48 residues, consisting of the coding L-amino acids, their D-counterparts, and analogs of natural amino acids, specifically norleucine, selenomethionine, ornithine, norvaline, l- and d-alpha aminobutyric acid, pyroglutamic acid, and the acetyl capping group.

Another largely unsolved issue is the generation of suitable 3D conformations for peptides that can be used as starting points for MD simulations [17], mutation pipeline analysis [18], or structure-based modeling and drug design. Currently, most tools either focus on structure prediction for whole proteins or small molecules, but they do not cover the middle occupied by medium- to large-sized peptides [5].

Finally, pharmaceutical companies generally require the all-atom representation of a molecule for its registration in various databases, and historically molecules have been drawn manually before registration. Even for short peptides such as the 9-mer oxytocin, manual drawing of these structures can be error-prone, and the rate of error grows as peptide molecules evolve to contain longer main chains, staples, cycles, or fatty-acid chains connected to a peptide main chain. There is a clear need for a platform that can take line formats of peptides, including arbitrary NNAAs, and generate correct atomistic representations with no manual intervention.

**Fig. 1:**
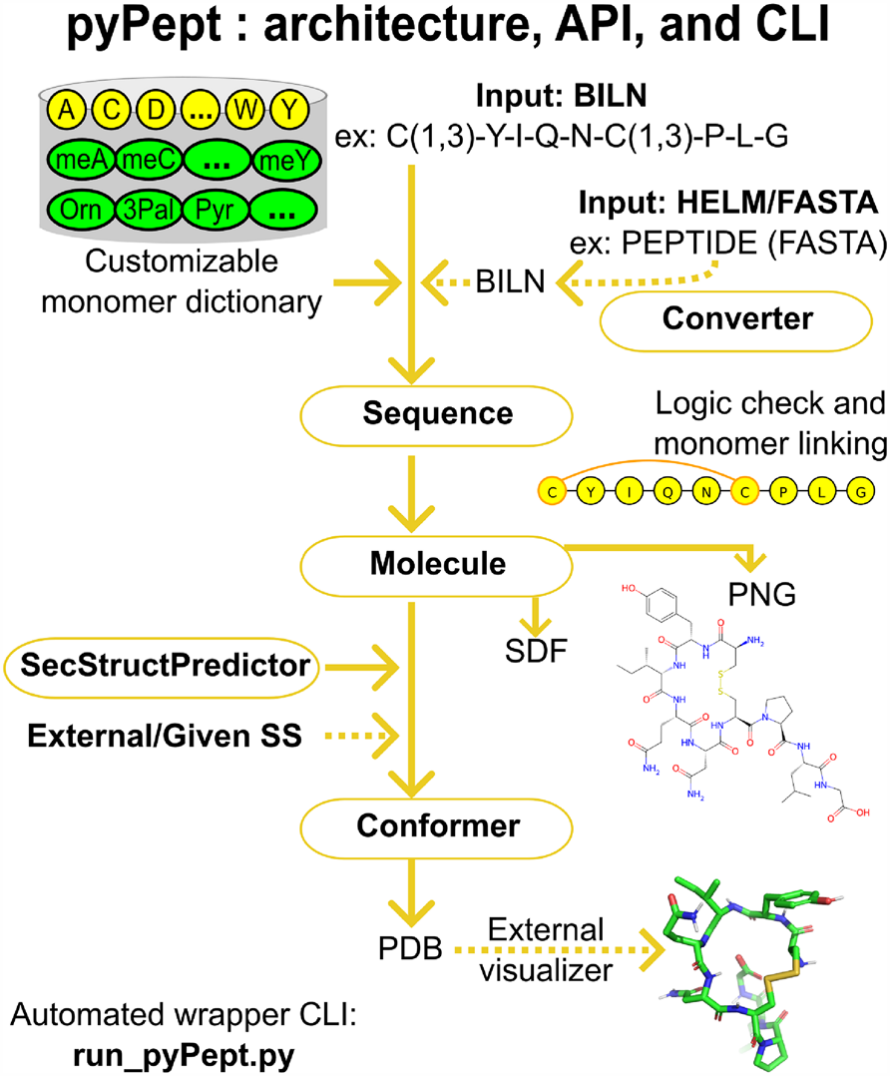
Summary of pyPept architecture and interfaces. Each monomer is mapped to chemical structure through a monomer dictionary, and monomers are connected by bonds defined for each monomer’s R-groups to yield a sanitized *Sequence* object. Information from the *Sequence* is used to create a *Molecule* object, and two options for 2D depictions are provided. Further, one can predict *Conformer* objects using additional secondary structure restraints. An executable driver program (run_pyPept.py) encapsulates the sequence-to-structure conversion, offering a non-programmatic way to obtain conformers directly from line notations. The solid lines indicate the default run_pyPept.py execution, with supported options shown by dotted arrows

To fill the void of a toolkit for working with peptides containing arbitrary monomers, unusual connections between monomers, or branching, we developed the python-language toolbox pyPept for handling complex peptides. pyPept is internally based on BILN, but it also can accept FASTA or HELM representations as input. Using a monomer library and the information contained in the input string, atom-level logical connections are validated, and the molecule can optionally be converted into a molecule object. This object can then be used to run typical cheminformatics analyses and predict conformers according to structural restraints. Figure 1 shows a general overview of the package.

Briefly, the package works as follows. The pyPept *Sequence* class converts the input line notation into a *Sequence* object, which is an ordered list of monomers together with all of the connectivity information necessary to accurately build the molecule. The *Molecule* class takes this *Sequence* object and creates a molecule object with a sanitized 2D representation. The *Conformer* class leverages distance geometry functionality to generate a 3D conformer. Here we found it necessary to provide secondary structure constraints in the 3D generation to obtain conformations that can be close to a bioactive one. Therefore, we developed a method to predict peptide secondary structure elements, which we packaged into the *SecStructPredictor* class. In addition, we developed a helper class *Converter* which can be used to translate from HELM to BILN and back, or to convert a FASTA string into BILN. A wrapper script (run_pyPept.py) is also provided that automates the sequence-to-structure/conformer conversion of a general peptide, thus demonstrating how to connect the individual components, and providing a non-programmatic way to use pyPept by simple command line execution.

## Requirements

pyPept has been written in Python 3.9 using language-standard internal libraries in conjunction with the external packages RDKit [16] and BioPython [19], both available through a package installer such as Conda. Instructions on how to install pyPept using a setup.py script are provided in the code repository. Examples of execution and direct module calls are given in the repository README file and in a directory “examples” included in the software distribution.

## Methods

### Secondary structure prediction

From the BIOLIP database (version 04.2022) [20], we extracted the 8112 bioactive peptides for which secondary structure annotations were returned by the DSSP software [21]. The peptides, composed of natural amino acids, are unique sequences showing a diverse set of possible bound conformations, including 30% of helical peptides and 10% forming parallel or anti-parallel beta sheets, even for small peptides of five or six amino acids. They were used to develop a matching algorithm between a query sequence and the bioactive conformers.

Our method compares the query peptide by matching its amino acids to those contained in database sequences, where a substitution matrix generates the matching score [22]. The selected matrix was fitted to capture the similarity between known protein structures and is available in the BioPython package [19]. We chose to not allow alignment gaps, thus, the comparisons are made between fragments of the same length. Therefore, if the query sequence is shorter than the database sequence, we compare it with fragments of the database sequence. Inversely, when the database sequence is shorter than the query sequence, the query sequence is fragmented to obtain peptides of the same length.

In practical applications, we recommend a peptide query length in the range of 5–20 amino acids, given that the reference set of bound peptides from BIOLIP has a maximum length of 30 amino acids. For each comparison between sequences *A* and *B*, we calculate a similarity score using the selected substitution matrix and normalize it by:

1 $$S_{AB}=\frac{score_{AB}}{\sqrt{score_{AA}*score_{BB}}}$$

where $score_{AB}$ is the alignment score between the two peptides, and $score_{AA}$ and $score_{BB}$ are the alignment scores for each peptide with itself.

After finding matches above a similarity threshold, a profile with the hits is created, and each amino acid in the query sequence is assigned the most frequent secondary structure element. Using a set of experimental peptide structures with different secondary structure categories, we found a threshold for $S_{AB}$ in the range of 0.6–0.7 to be suitable (see Additional file 1).

We also compared the predictions of our method against various state-of-the-art tools such as PSIPRED [23], ModPep [24], and AlphaFold2 [25]. Specifically, we selected a list of 38 peptides available in the PDB with lengths between 8 and 17 amino acids and a diverse set of secondary structure motifs to test the predictions (see Additional file 1: Table S2). We found that the approach described here correctly predicts the secondary structure for most peptides, with 8 of 10 correct predictions for complex motifs based on co-occurring alpha-helix and beta-sheet conformations. This result was on par or better than the methods tested (see Additional file 1: Table S3). Our method can be easily embedded with the rest of the pyPept functionalities. Still further, the user can include secondary structure restraints from any other method or simply by manually providing the expected conformation as exemplified in the “Typical workflow” section.

### Monomer library

Allowing arbitrary monomers in a peptide sequence affords monomer definitions that connect the identifiers in the line notation with the underlying chemical structure. In pyPept, we use a dictionary which, for each monomer, contains the following information:

- chemical structure
- connection points
- possible leaving groups (that are removed when the respective connection points are used in a bond between monomers)
- the abbreviation to be used in the peptide line notation
- the natural analog of a monomer, if applicable
- additional information about a specific monomer such as its role, i.e., amino acid or capping group,
- its stereochemical SMILES representation, and finally,
- a corresponding PDB residue code.

As per the extensible definition of BILN [14], these monomers can be any non-natural amino acid or non-amino acid with annotated leaving groups that will allow the formation of inter-monomer bonds. No assumptions on the type or nature of monomers, or their connections, are made. This allows the formation of additional bonds to describe branched peptides, as well as cyclic peptides using peptide, disulfide, or potentially other types of bonds (see examples in Table 1).

The Pistoia Alliance maintains a dataset of 322 HELM monomers [26]. This dataset closely follows the monomer entry (dictionary) format described above; thus, the 322 HELM monomers are adaptable for use in pyPept with only minor conversion effort. For convenience and to remove an entry barrier to apply pyPept, we provide a python script in the repository to convert the HELM monomer dataset into a format suitable for pyPept, as well as a structure definition file (SDF) with this modified monomer information. This can also be used to add proprietary monomers, as long as they are provided as a SDF file in the Pistoia monomer format. Specifically, the user can add the SDF format of the new monomer in the monomers.sdf file, which is located in the “data” folder of pyPept. The SDF requires some tags to allow the correct mapping into the dictionary, including the name of the monomer, the type of monomer (amino acid or capping group), the abbreviated symbol, if the monomer has a natural analog, and the corresponding leaving R-groups to bond other monomers. An example of a monomer entry using the SDF format is provided in the data folder with the name example_preProcessed_monomer.sdf.

We note that in the Pistoia monomer set, no PDB residue names are provided. We chose to use the names reported in the chemical component dictionary [27]. If a monomer is not contained in this dictionary, a new random, though nonetheless unique, PDB code is created.

## pyPept design: key classes and examples

### Sequence class

This is the main class of the pyPept package. It converts the input BILN string (or HELM/FASTA transformed by the *Converter* class) into a *Sequence* object. In Table 1 we show some examples of peptides using the three input formats, where the FASTA format can be used only to represent natural amino acids, and it includes no information on branching or cyclization.

**Table 1:** Examples of peptides using the three input formats BILN [14], HELM [13], and FASTA

BILN	HELM	FASTA
P-E-P-T-I-D-E	PEPTIDE1{P.E.P.T.I.D.E}$$$$V2.0	PEPTIDE
ac-D-T-H-F-E-I-A-am	PEPTIDE1{[ac].D.T.H.F.E.I.A.[am]}$$$$V2.0	None
C(1,3)-A-A-A-C(1,3)	PEPTIDE1{C.A.A.A.C} $PEPTIDE1,PEPTIDE1,1:R3-5:R3$$$V2.0	CAAAC
C(1,1)-A-A-A-C(1,2)	PEPTIDE1{C.A.A.A.C} $PEPTIDE1,PEPTIDE1,1:R1-5:R2$$$V2.0	CAAAC
A-G-Q-A-A-K(1,3)-E-F-I-A-A.G-L-E-E(1,3)	PEPTIDE1{A.G.Q.A.A.K.E.F.I.A.A}| PEPTIDE2{G.L.E.E} $PEPTIDE1,PEPTIDE2,6:R3-4:R3$$$V2.0	None
N-Iva-F-D-I-meT-N-A-L-W-Y-Aib-K	PEPTIDE1{N.[Iva].F.D.I.[meT].N.A.L.W.Y.[Aib].K} $$$$V2.0	None

BILN’s support for specification of R-groups in bond formation means that linkage types can be easily specified. The BILN notation uses the monomer format *m(n,i)* to indicate that monomer *m* is a part of the cycle or branch assigned ID number *n* and connects via R-group *i* to a paired monomer *p(n,j)*. *i* and *j* can be any R-groups involved in single bond linkage formation. Thus, the cyclization by cysteine linkage [C(1,3)] is by disulfide bond in the third example but by peptide bond [C(1,1), C(1,2)] in the fourth example

The *Sequence *object holds a list of dictionaries, with each dictionary containing the necessary information for one monomer in the peptide sequence (see above, Monomer Library). In addition, a *Sequence* object stores the information about which monomers are connected and which atoms form these bonds. A *Sequence* object goes beyond a pair of bonds found in two adjacent amino acids of a linear FASTA sequence and also manages the information necessary for cycles, branches, staples, and other peptide-specific bond structure.

In all monomer structures, the R-groups at connection points that are not involved in bonds are replaced by their correct leaving group (e.g., the R2-group at the C-terminal end of the peptide is replaced by an OH forming the C-terminal carboxylic acid). During this procedure, checks guarantee that the input BILN string is not malformed, that the correct number of bond identifiers are present, and that it only contains monomers included in the monomer library.

As a final processing step, we change the names of the atoms that are part of an amino acid residue and those of the capping groups to follow the IUPAC convention which appends greek letters to the element symbol (e.g., C$\alpha$ as CA, C$\beta$ as CB, hydrogens HB2 and HB3 attached to C$\beta$). To achieve these changes, the greekify method from the rdkit-to-params package [28] was adapted for our needs.

A class method reads the monomer information and stores it in a Pandas DataFrame [29] object to allow easy access for the various *Sequence* methods.

### Molecule class

The *Molecule* class contains methods to convert the *Sequence* object into an RDKit ROMol molecule object. To accomplish this, we sequentially take each monomer in the *Sequence* object, merge its RDKit representation with the growing peptide and then add, if applicable, the appropriate bond(s) between the new monomer and the peptide.

To obtain an extended conformation of the peptide without overlapping atoms, the rdDepictor module from RDKit is used [30]. Alternatively, we have developed a procedure which changes the phi/psi angles in the protein backbone to obtain an extended 2D conformation and adjusts the torsion between C$\alpha$ and C$\beta$ to obtain an aesthetically pleasing 2D depiction of the peptide without overlapping atoms (see Fig. 1 for an example).

At this point, the 2D peptide object can be exported by a *Molecule* object method to different molecular formats, such as SMILES or SDF.

### SecStructPredictor class

Initial tests showed that the inclusion of secondary structure information is necessary to have a chance of obtaining a 3D structure that is close to the experimental conformation and is suitable for 3D modeling tasks. As this experimental information is often unavailable, and the existing secondary structure prediction tools did not return results sufficiently accurate enough for our purposes when applied to short and medium-length peptides, we decided to develop a similarity-based tool to assign secondary structure motifs to the peptides based on a dataset of bioactive conformers available in the PDB (see Methods section).

The *SecStructPredictor* class collects the functionality to obtain, for a given peptide, a prediction of its secondary structure. Since experimental peptide structures are mostly of natural amino acids, in this protocol, non-natural amino acids are first mutated into their natural analogs, then this mutated peptide is compared with all sequences in the database. To assign the natural analog, pyPept checks first if the information about a natural analog was included in the dictionary. If not, a fingerprint-based similarity run is performed between the monomer of interest and the 20 standard natural residues. A potential natural analog is assigned based on the highest Tanimoto score above a threshold of 0.5. Otherwise, the non-natural amino acid is replaced by an alanine.

After this mapping and search for matching contexts, the secondary structure element for each residue in the original peptide is returned. The secondary structure categories are: B (beta bridge), H (alpha helix), E (beta strand), S (bend), T (turn), and G (3/10 helix). Of course, any other secondary structure prediction tool can be used to generate these annotations and use them to drive pyPept’s conformer generator.

### Conformer class

The *Conformer* class is used to generate a 3D conformer of the peptide. We employ the ETKDGv3 (Experimental-Torsion Knowledge Distance Geometry) method from RDKit followed by minimization of the structure [31, 32].

Using distance geometry without any constraints usually leads to random coil 3D structures. To end up with peptide conformations that are helical, for example, one needs secondary structure information as constraints for the algorithm. As this information often is not available experimentally, we suggest to use a tool to predict the peptide secondary structure. This can be the *SecStructPredictor* class presented above, or any other method.

Based on the input secondary structure elements, fixed distances are assigned in the RDKit-defined distance bounds matrix to force the formation of $\alpha$-helix or $\beta$-sheet conformations, which is not a feature available in small molecule-oriented packages such as RDKit. The constraints are complemented by the ETKDGv3 knowledge-based potential to predict the peptide conformers. In the case of non-natural amino acids, the natural analogs (if available in the monomer dictionary) are used to assign the secondary structure element. If no natural analog is available, alanines are used instead. At the end of this processing pipeline, a PDB file can be generated with unique 3-letter residue codes and atom names conforming to the IUPAC rules.

In our experience, this procedure is suitable for sequences shorter than 20 amino acids; for longer sequences, many well-established protein modeling tools are available as well [33].

We note that AlphaFold [25, 34] can also predict the conformations of even short peptides, which are often surprisingly close to the experimental bound or free structure. However, this is again a tool that can only deal with natural amino acids. Thus, pre- and post-processing steps are necessary: first, replace the NNAA with a close natural analog; second, conduct the AlphaFold prediction; third, mutate the analog monomer back to the NNAA using the conformer obtained.

### Converter class

The native input format of the *Sequence* class is BILN. To allow one to start from a HELM or FASTA representation, we also provide a format conversion class [14]. The *Converter* class allows a two-way conversion between HELM and BILN, and from FASTA to BILN.

## Typical workflows

### API-based workflow

**Fig. 2:**
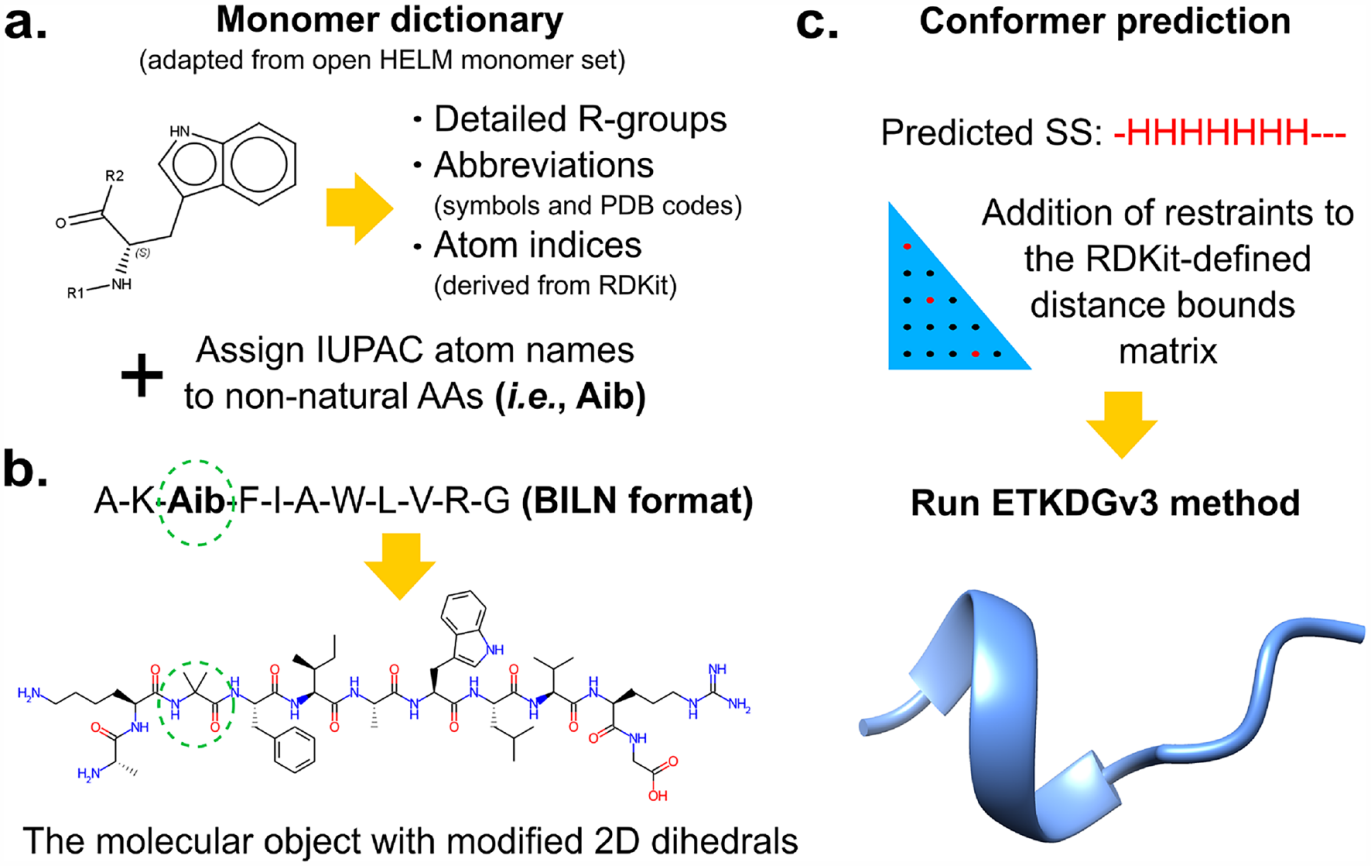
Detailed description of peptide 2D/3D generation from sequence. **a** Main components of the monomer dictionary used to define each BILN component and to allow the generation of peptide bonds between them. In addition, the monomer atoms are named according to the IUPAC convention. **b** Example of a peptide with a non-natural amino acid and the 2D depiction of the RDKit molecular object with modified peptide bond dihedrals to minimize overlapping atoms. **c** Scheme showing the prediction of the secondary structure of the example peptide in (**b**), the addition of restrained distances into the RDKit bound matrix, and the subsequent prediction of the most probable conformer using the ETKDGv3 method

We envision a typical use case in which one wishes to obtain a 2D representation stored in SDF format, starting from a BILN sequence. With the aforementioned pyPept classes, this could look as follows:
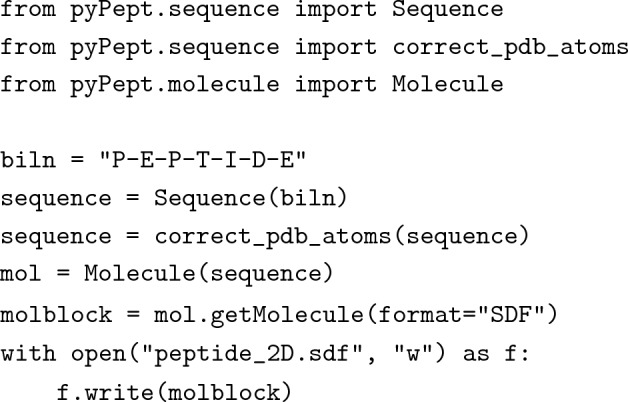


From there, a few lines of additional code would then generate a PDB file with a 3D representation of the input peptide, based on the prediction or specified input of its secondary structure:
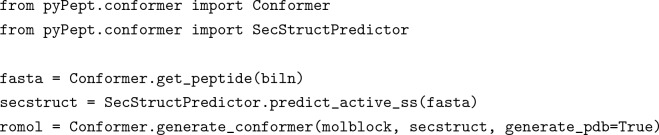


A graphical summary of this workflow is shown in Fig. 2.

The workflow above is a routine task. To automate this workflow and remove the need for one to implement it themselves, we provide a command line wrapper script which takes the peptide representation and additional options as command-line arguments:
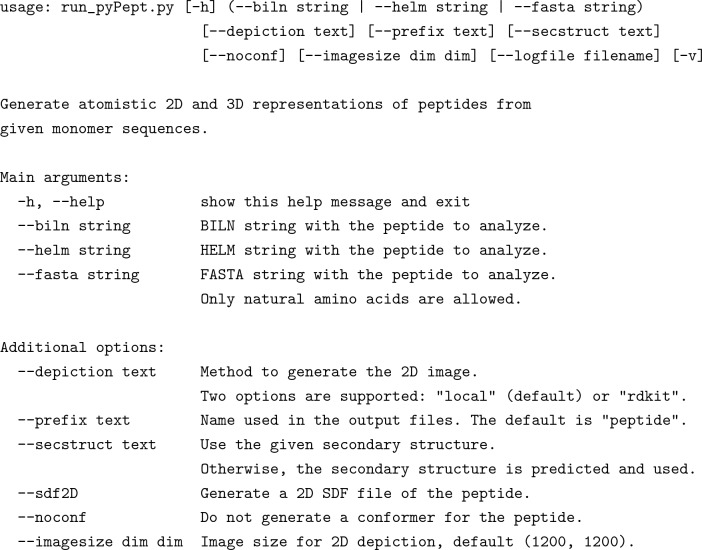


### All-in-one execution

**Fig. 3:**
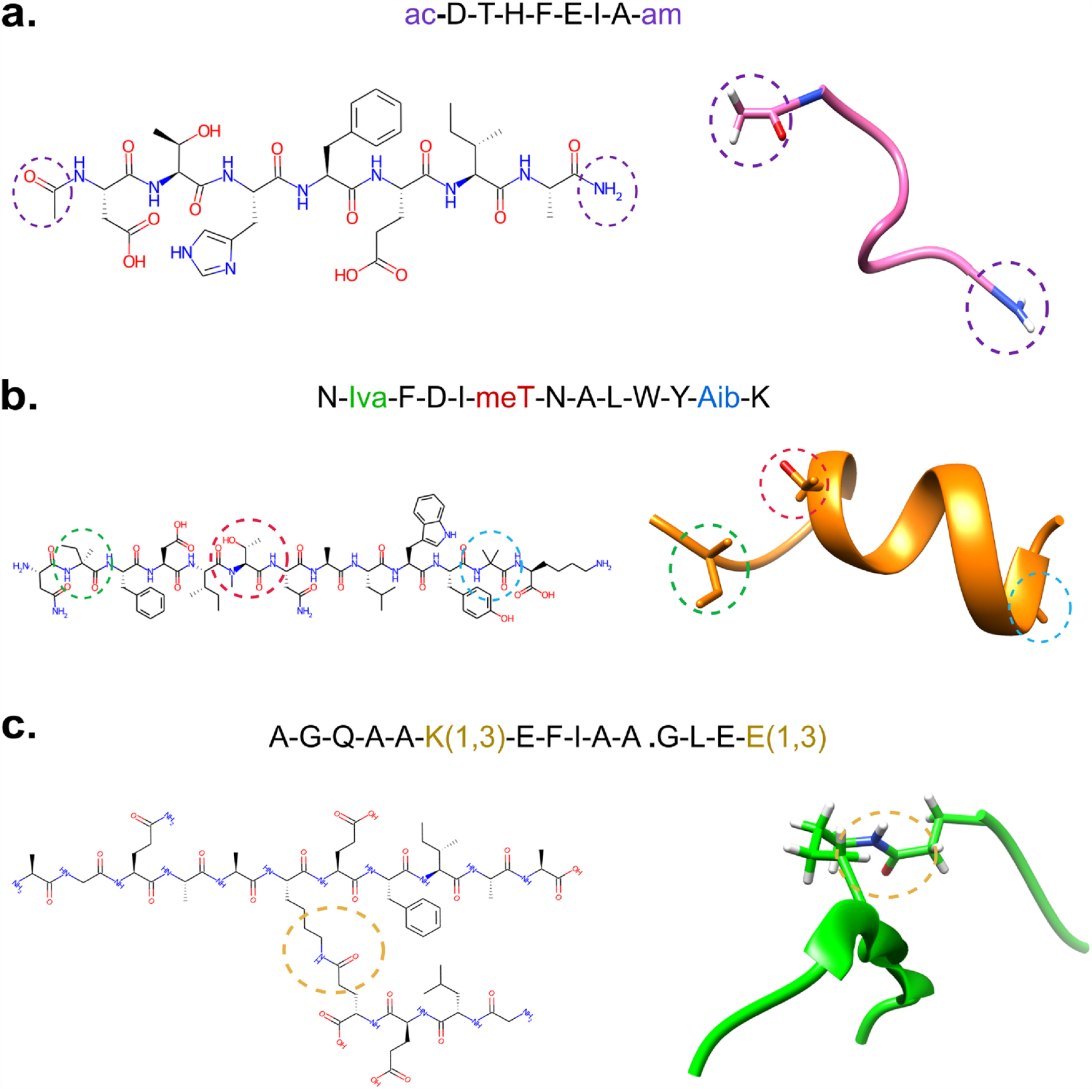
Example of peptides formatted with pyPept, sequences are shown in BILN format. **a** Capped peptide with acetyl group at the N-terminal part and an amino group at the C-terminal part. **b** A peptide with three non-natural amino acids highlighted in green (Iva: Isovaline), red (meT: *N*-Methyl-Threonine) and blue (Aib: Alpha-aminoisobutyric acid). In this case, the main peptide was predicted as an $\alpha$-helix. **c** A peptide with a branch generated between a lysine and a glutamic acid through the third R-group located in their side chains. The bridge is identifiable in both the 2D and 3D representations

The sequence-to-conformer protocol can be run all at once by executing the provided wrapper script. An example execution using a randomly-generated peptide sequence is as follows:
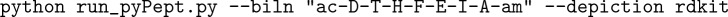
where the capped peptide in BILN format is used as input (with quotation to avoid any mis-processing by the host operating system), and the RDKit built-in function is used to generate the 2D depiction. As examples, we ran the method with a set of peptides having different features, including the presence of non-natural amino acids, capping groups, and the presence of multiple chains (Fig. 3). In the second case (Fig. 3b), the peptide main chain is predicted as partly $\alpha$-helical based on our conformer prediction method. In the third case (Fig. 3c), a branched peptide with a bond connection between a lysine and a glutamic acid is shown.

## Conclusions

With clinical precedent for their therapeutic benefit, peptides have been and will continue to be actively developed, with increasingly complex topologies, necessitating a complementary infrastructure for peptide information communication (e.g., BILN in presentations or patent applications), automated conversion of the human-communicable format into formats that can be directly submitted to compound registration systems, and for computational chemistry purposes. The pyPept package provides a publicly accessible collaborative effort to achieve these goals, with a low barrier to entry which enables less tech-savvy experimental and design research organizations to maximally benefit. pyPept facilitates the generation of 2D and 3D conformations of a peptide even in the presence of non-natural amino acids, non-amino-acid monomers, branches, and cyclic structures, which are certain to increase as peptide synthesis technologies have continued to improve.

For peptide design teams, they can easily convert a series of peptides stored in a spreadsheet with one monomer per column into matching BILNs. Then, it is straightforward to directly apply the pyPept 2D depiction pipeline and generate 2D representations for all peptides. Since 2D representation is still at the core of the compound registration process for many companies, use of pyPept for systematic representation generation avoids the error-prone manual drawing of peptide structures.

The 3D pipeline produces a peptide structure that can be used as a starting point for MD simulations, structure-based modeling efforts, or other methods to obtain low-energy conformations of the peptide [35]. It remains to be clarified, admittedly, how well our procedure predicts the bioactive conformations of peptides. One of the issues is that all secondary structure predictors (as other peptide/protein conformer predictors, including AlphaFold) work based on natural amino acids. The introduction of NNAAs in a post-processing step may completely alter the local conformation and, thus, the overall structure of the peptide. We fully acknowledge the need for a more systematic analysis.

Despite such an aspect, we believe that pyPept is a framework that will facilitate the generation of 2D and 3D structures of complex peptides, reducing human error and accelerating not only drug discovery but all research fields involving peptides.

### Data availability

- Project name: pyPept (version 1.0)
- Project home page: https://github.com/Boehringer-Ingelheim/pyPept
- Operating system(s) tested: Linux
- Programming language: Python 3.9 or higher
- Other requirements: RDKit 2020 or later; Biopython 1.7.9 recommended.
- License: MIT

The code is available as a Github repository. Any questions related to the implementation can be directed to the authors’ email accounts.

### Supplementary Information

**Additional file 1.** Validation and assessment of the secondary structure predictor.
