# Supplementary material for: pyPept: a python library to generate atomistic 2D and 3D representations of peptides
Source: J Cheminform. 2023 Sep 12;15:79. doi: 10.1186/s13321-023-00748-2 (PMC10498622; doi:10.1186/s13321-023-00748-2)
Supplement: Supplementary file 1 — Additional file 1. Validation and assessment of the secondary structure predictor. [file 13321_2023_748_MOESM1_ESM.pdf]

Supplementary Information: pyPept: a python library to generate atomistic 2D and 3D representations of peptides

Rodrigo Ochoa, J.B. Brown, Thomas Fox

Medicinal Chemistry, Boehringer Ingelheim Pharma GmbH & Co KG, 88397 Germany

1 Validation of secondary structure predictor

To validate the protocol, we generated three query sequence subsets: 10 helical peptides, 10 anti-parallel sheet peptides and 10 coils. We predicted the secondary structure motifs of the query peptides using the known secondary structure database with 8-mer fragments at different composition percentages: 100, 90, 80, 70 and 60. The database subgroups were selected randomly, and 10 independent runs per subgroup and query peptides were calculated. Based on that, we generated a final average score by comparing position by position the predicted secondary structure elements with the experimental data. In that way, we can check how dependent is the similarity metric to the database size and the random selection of sequences.

Table 1: Sequences used to validate the similarity-based algorithm to predict bioactive secondary structure motifs in peptides shorter than 30 amino acids. A peptide id, the sequence and the experimental secondary structure is reported. The secondary structure elements means: B (beta bridge), H (alpha helix). E (beta strand), S (bend),T (turn) and G (3/10 helix).

Id	Sequence	Secondary structure
helix-pep1	WFSKLASSAFSGLFGALLA	-HHHHHSS—S-SS—-
helix-pep2	GSSFLSPEHQRVQQR	——TTHHHHTT-
helix-pep3	RGTEAAKKKYAQVCVTMPTAKICRY	–SSSHHHHTHHHHHHSTT-HHHH-
helix-pep4	EVVKLLLEAGADVNAQDK	-HHHHHH——SS—
helix-pep5	AENLYQIIGI	-HHHHHHHT-
helix-pep6	PKFGTHHKALQEIRNSLLPFANE	-TTGGGHHHHHHHHHHHGGG—
helix-pep7	VAAMAFGHVIGVAIVLGLT	–HHHHHHHHHHHHHHT–
helix-pep8	METITYV	–HHHH-
helix-pep9	SFKEELDKYF	-HHHHHHHH-
helix-pep10	TILFQLALAALVILSFVMVIGVPVAYAS	-HHHHHHHHHHHHHHHHHHHHHHHHTTT
sheet-pep1	KSIRIGPGQAFYAT	–EEETTTTEEEE-
sheet-pep2	RIHIGPGRAFYTTK	-EEETTTTEEEE–
sheet-pep3	KRIHIGPGRAFYT	—EETTTTEE–
sheet-pep4	DENLPEWAIENPSKLGGSFDASGAFHG	—–STTTS-S—–EE-TTS-EE-
sheet-pep5	YDLMLVHTNRHYGKTLVLNMQT	–EEEE–TTTTT–EEEES–
sheet-pep6	REEIGTVIDHVEGLPSVM	—–EE—EE–TT–
sheet-pep7	TISAADIEGAIEDYVSSFS	—–EE—EE–TT–
sheet-pep8	QHLGDSLVRC	-EEETTEE–
sheet-pep9	GFCQRPICFPN	-EE—-EE–
sheet-pep10	EIGTVIDAAHVEGLPSV	—EEEE–EEE–TT-
coil-pep1	ILFPSSERLISNR	–S-SSSS—–
coil-pep2	PAAKRVKLD	———
coil-pep3	KRVKL	—–
coil-pep4	AVKRPAATKKAGQAKKKKL	——SS—–S—–
coil-pep5	SRDHSRTPM	—-S—-
coil-pep6	SAPDTRPA	——–
coil-pep7	EPCCDSCRCTKSIPPQCHCANI	–S-SS—–SSS—–S–
coil-pep8	EPGPYAQPSVNTK	—SS——–
coil-pep9	SSVIGVWYL	——S–
coil-pep10	RKSLTIYAQVQK	————

After multiple trials with peptides from different secondary structure categories, we obtained consistent prediction results with matching percentages above 70% for most of the sequences, even after reducing randomly the training set to a 60% of its original size (see Supplementary Figure 1).

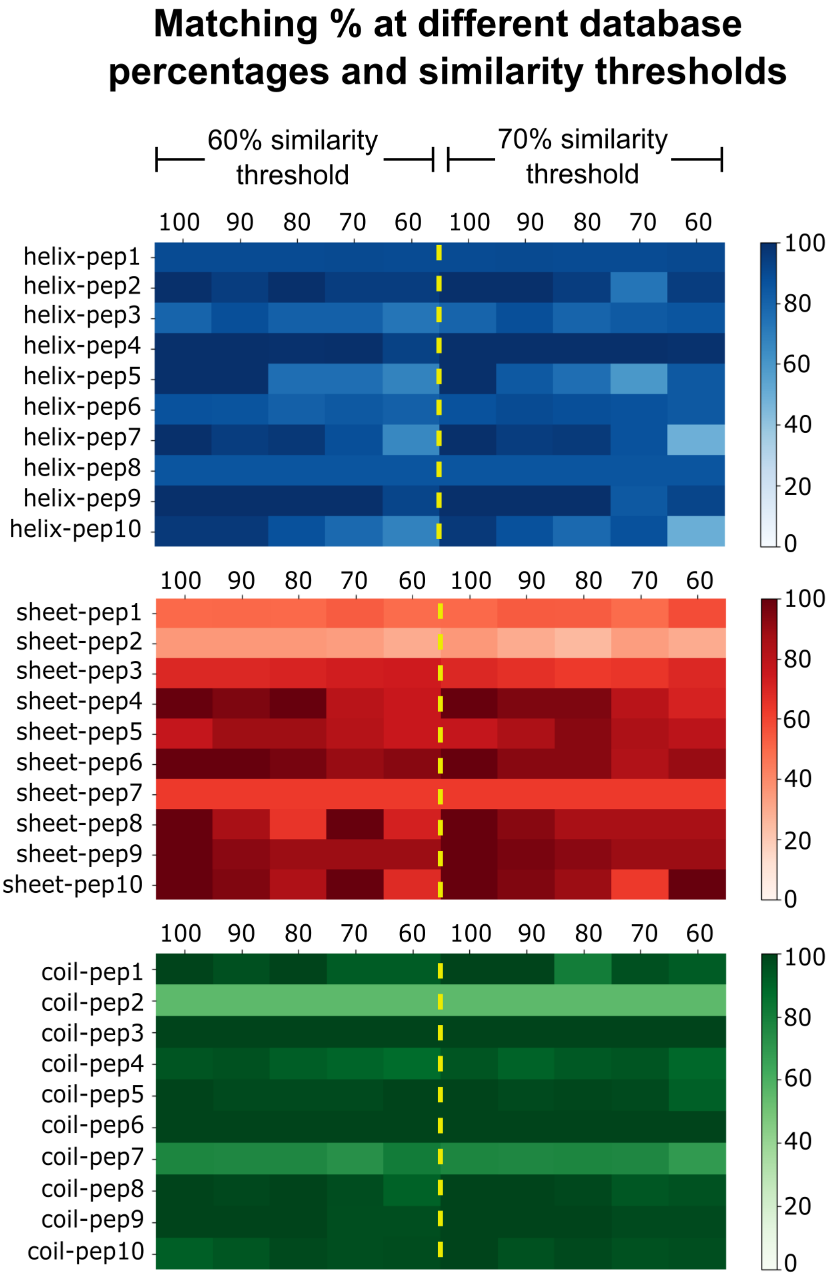

Figure 1: Validation of the similarity prediction method. Three representative sets of 10 peptides were chosen, composed of 10 alpha-helices (blue), 10 beta-sheets (red) and 10 coil peptides (green). Each heatmap is split in two parts. In the left a similarity threshold of 60% was used to find similar hits, and in the right a threshold of 70%. Each part is split into 5 columns that represents the percentage of the peptide secondary structure database used to run the similarity analysis. The color in the heatmap accounts for the average match similarity between the experimental secondary structure motif and the predicted secondary structure elements. The darker, the more similar. The analysis look for evaluate the impact of reducing randomly the peptide database as a measure of the matching robustness.

2 Assessment of the secondary structure predictor

To evaluate the impact of our internal secondary structure prediction method, we selected a set of peptides available in the PDB with lengths between 8 and 17 amino acids, and with a diverse set of secondary structure motifs to test the predictions. A list of 38 peptides were included and shown in Supplementary Table 2.

Table 2: List of peptide sequences used to assess the secondary structure prediction methods. The sequence, internal id, PDB id and sequence length are provided.

Sequences	Id	PDB id	Length
SFEGYDNS	pep1	5d0j	8
KLDPIIED	pep2	5jja	8
AARKSAPA	pep3	2g46	8
GVLKEYGV	pep4	5ojo	8
GDQNATFG	pep5	3rce	8
KILHRLLQ	pep6	1bsx	8
EERIISLD	pep7	6v7r	8
NLVPMVATV	pep8	2x4r	9
SRWRFPARP	pep9	3u85	9
FAPGNWPAL	pep10	4pgb	9
MCLRMTAVM	pep11	5e8n	9
TARKSTGGW	pep12	3zg6	9
YSNTLPVRK	pep13	6bqt	9
MSQIKRLLS	pep14	2jkr	9
ARTKQTARY	pep15	6hbo	9
GLLDALDLAS	pep16	2c23	10
CPAYSAYLDC	pep17	4x1n	10
EEFELLISNS	pep18	5jer	10
RAHSSPASLQ	pep19	3mhr	10
GEAEERIISL	pep20	6v7r	10
GPRLSRLLSYA	pep21	1om2	11
QGLLDALDLAS	pep22	2o02	11
DFEEIPEEYLQ	pep23	1c1u	11
SQFDLSTRRLK	pep24	5icy	11
QGLPPRPKIPP	pep25	4apj	11
GEAEERIISLD	pep26	6v7p	11
AEERIIVLSDSD	pep27	4wjq	12
PRLSRLLSYAGC	pep28	2v1s	12
KLMFKTEGPDSD	pep29	6rjz	12
NNRDPIVISDSP	pep30	6jxu	12
CFTARMSPPQQIC	pep31	4z09	13
PPGGRGLTGPIGP	pep32	5mv3	13
GVYATRSSAVRLR	pep33	6atf	13
DIFERIASEASRL	pep34	6biy	13
NWFDITNWLWYIKK	pep35	2fx7	14
APSTGGVMKPHRYR	pep36	5jjy	14
KETAAAKFERQHLDS	pep37	1fev	15
LETLAPYIPMDGEDFQC	pep38	6i7r	17

We also predicted the secondary structure using three external tools: PSIPRED, which is focus on predicting secondary structure motifs for proteins; ModPep, a web server to predict 3D structures of peptides; and AlphaFold2, a deep learning modelling tool to predict 3D structures of proteins using the amino acid sequences. We compared the experimental secondary structure with the output of each tool based on the conservation of main motifs such as α-helix and β-sheets conformations (Supplementary Table 3).

Our method performs similarly or even better than some of the state-of-the-art tools, with 8 of 10 complex SecStr motifs assigned correctly. However, any of the assessed methods are suitable options to implement, but with some limitations in terms of massive analysis in the case of the web servers, or computational demand regarding the 3D conformer prediction approaches. Our *in-house* approach is a similarity-based alternative that can be integrated easily with the other pyPept modules as illustrated in the main manuscript.

Table 3: Predicted secondary structure for a set of peptides calculated using our internal secondary structure prediction, and the methods:, PSIPRED, ModPep, and AlphaFold2. The ids are associated to the peptides from Supplementary Table 2. The SecStr elements means: B (beta bridge), H (alpha helix). E (beta strand), S (bend), T (turn) and G (3/10 helix).

Ids	Experimental	Internal	PSIPRED	ModPep	AlphaFold2
pep1	–TTS—	–TTS—	———	—-SS–	———
pep2	———	———	———	–SS-S–	———
pep3	———	———	———	———	———
pep4	—GGGG-	———	———	–SGGG–	———
pep5	–SS—-	–SS—-	———	—-SS–	———
pep6	–HHHHH-	-HHHHHH-	-HHHHHH-	—-TT–	–GGGGT-
pep7	———	———	———	-TT——	———
pep8	—S——	—S——	——EEE-	—-S—-	————
pep9	—-STT–	——TT–	————	–SSS-S–	————
pep10	—-S—-	————	————	—STTT–	—-S-TT-
pep11	——S–	——S–	——EE-	—-SS—	–TTSTT–
pep12	——TT-	————	————	–TT——	————
pep13	—-SS—	-SS—S–	————	–SSS-S–	————
pep14	–TTS-S–	-HHHHHHH-	-HHHHHHH-	—-S—-	–GGGGT–
pep15	————	————	————	—TTSS–	————
pep16	-HHHHTT—	-HHHHTT—	–HHHHHH–	—-SS—-	–SHHHHTT-
pep17	——TT-	–TT—TT-	————-	—S-TTT–	–SS———
pep18	————-	————-	—EE——	-TTHHHHS–	-HHHHHHHT-
pep19	——S—	——S—	————-	—SS-SS–	————-
pep20	————-	————-	–HHHHH—	–SSS——	————-
pep21	——SGGGG-	-GGGHHHHHH-	–HHHHHHH–	–S-S-SSS–	–SSTTTTTT-
pep22	-HHHHHTT—	-HHHHHTT—	–HHHHHHH–	—SSSS—-	-HHHHHHHHT-
pep23	——GGGT-	——GTG–	-HHH–HHHH-	—-S-STTT-	——GGG–
pep24	–EETTTTEE-	-EEETTTTEE-	—————	—-SSSS—	—————
pep25	–S————	—————	—————	——S—-	—————
pep26	—————	—————	–HHH——	–SSS——	—————
pep27	——————	——————	—EEEEEE—	-HHHH—SS–	——————
pep28	–HHHHHHHTT-	–HHHHHHHTT-	–HHHHHHH—	—HHHHHH—	—SSSSTT—
pep29	——SS—-	——SS—-	——————	–S-SSSS—-	——————
pep30	——————	——————	——————	——S-SSS–	——————
pep31	-EE–SSS–EE-	-EE-SSSS–EE-	——————-	—-SSSS–S– -EEE-SSS-EEE-	
pep32	–GGG-SSS—-	—-S-S——	——————-	—S——S—	——————-
pep33	——————-	——————-	——————-	-HHHHT–SSS–	————TT–
pep34	——————-	——————-	-HHHHHHHHHHH-	–HHHHHHHTS–	-HHHHHHHHHHH-
pep35	-GGGHHHHHHHHH-	-HHHHHHHHHHHH-	——HHHHEEE–	–S—-SSSSS–	-HHHHHHHHHHHT-
pep36	–SS————-	———————	———————	—SSS–SS—-	—-TT———
pep37	—HHHHHHHHH—	—HHHHHHHHH—	-HHHHHHHHHHHH–	–S-SSS-THHHH–	—HHHHHHHHHS–
pep38	————TT———	————T———-	————————	—TTT—-SSSSS–	————-S——
